# Vitamin K2 Improves Osteogenic Differentiation by Inhibiting STAT1 via the Bcl-6 and IL-6/JAK in C3H10 T1/2 Clone 8 Cells

**DOI:** 10.3390/nu14142934

**Published:** 2022-07-18

**Authors:** Huakai Wang, Longxian Li, Nan Zhang, Yongxi Ma

**Affiliations:** State Key Laboratory of Animal Nutrition, College of Animal Science and Technology, China Agricultural University, Beijing 100193, China; huakaiwhk@cau.edu.cn (H.W.); s20193040586@cau.edu.cn (L.L.); s20203040629@cau.edu.cn (N.Z.)

**Keywords:** osteoporosis, vitamin K_2_, differentiation, C3H10 T1/2 clone 8 cell

## Abstract

Osteogenic activity of vitamin K_2_ (VK_2_), a small molecular nutrient, has been suggested. However, the underlying mechanisms have not been fully elucidated. Therefore, this study aimed to explore the mechanisms by which VK_2_ promotes osteogenic differentiation. The effects of VK_2_ on osteogenic differentiation indicators were determined in C3H10 T1/2 clone 8 cells. The RNA-seq analysis was used to explore the hypothesis that VK_2_ promotes osteogenic differentiation. Small interfering RNA (siRNA) assay and plasmid transfection assay were used to determine the potential role of VK_2_ in the modulation of Bcl-6/STAT axis and IL-6/JAK/STAT signaling pathway. VK_2_ significantly increased alkaline phosphatase (ALP) activity, ALP, osteocalcin (OCN), and RUNX2 abundance, and RUNX2 protein expression. RNA-seq analysis showed that there were 314 differentially expressed genes (DEGs) upregulated and 1348 DEGs downregulated by VK_2_. PPI analysis determined the top 10 hub genes upregulated or downregulated by VK_2_. Overexpression of Bcl-6 increased osteogenic differentiation and decreased expression of STAT1. Administration with VK_2_ restored the inhibition by siBcl-6 in osteogenic differentiation. Knockdown of IL-6 decreased the mRNA levels of genes associated with the JAK/STAT signaling pathway, and increased markers of osteoblast differentiation. Furthermore, treatment with VK_2_ improved inhibition in osteogenic differentiation and decreased enhancement of JAK/STAT signaling pathway related genes by overexpression of IL-6. Our study suggests that VK_2_ could improve osteogenic differentiation via the Bcl-6/STAT axis and IL-6/JAK/STAT signaling pathway.

## 1. Introduction

Osteoporosis is a common metabolic bone disease caused by an imbalance between osteoblasts and osteoclasts, which is characterized by reduced bone mineral density and bone quality [1,2]. The number of individuals suffering from high risk of osteoporotic fracture worldwide in 2040 is expected to reach >300 million due to continued increase in the relative proportion of the aging population, which is expected to bring substantial economic burden to society [3]. Currently, the drugs used to treat osteoporosis are mainly anti-resorption agents, which have a low treatment rate (19%) following an osteoporotic fracture [4]. The alternative therapy approach using osteoanabolic drugs is limited to three drugs, teriparatide, abaloparatide, and romosozumab [5]. However, the anti-resorptive or osteoanabolic drugs were restricted for use due to their toxic side effect. Therefore, it is imperative to identify and test for new therapeutic targets or drugs that promote bone anabolism without side effects.

Vitamin K (VK) is a class of fat-soluble vitamins that structurally contains 2-methyl-1, 4-naphthoquinones, including phylloquinone (K_1_), menaquinones (K_2_), and menadione (K_3_) [6]. It is well known that VK is a critical factor in the process of blood coagulation. In addition, VK is important at play in a wide range of biological activities, including cell growth and proliferation, inflammatory reaction, oxidative stress, and regulation of calcium metabolism in tissues [7]. In recent years, VK_2_ has become a research hotspot for scientists due to its osteogenic activity. Certain experiments have illustrated that VK_2_ plays an important role in the proliferation and differentiation of osteoblasts, while increasing the alkaline phosphatase (ALP) activity, osteogenic mineralization, and gene expression levels of osteogenic differentiation, such as ALP, osteoprotegerin (OPG), in pre-osteoblast cell line MC3T3-E1 [8,9]. Clinically, menaquinone 4 (MK4), the most common form of VK_2_ has been used to treat osteoporosis in Japan [10]. However, Binkley et al. reported that no effect of VK_2_ on lumbar spine or proximal femur bone mineral density or proximal femur geometric parameters was observed in healthy postmenopausal North American women [11]. In contrast, a meta-analysis from Cockayne et al. concluded that supplementation with VK_2_ reduces bone loss and incident fractures [12]. Furthermore, we have previously shown that VK_2_ improves bone microarchitecture in ovariectomized mice [13]. Based on the above, we speculate that VK_2_ could improve osteoblast differentiation and maintain bone health. However, the mechanism that VK_2_ promotes osteogenic differentiation remains unknown.

C3H10 T1/2 clone 8 cell is a mesenchymal stem cell line with multifunctional differentiation potential, which has been used to study the differentiation mechanism of mesenchymal cells into myocytes, adipocytes, and osteoblasts [14,15,16]. Therefore, we performed this study to evaluate the effects of VK_2_ on the osteogenic differentiation and to investigate the possible molecular mechanism involved in C3H10 T1/2 clone 8 cells.

## 2. Materials and Methods

### 2.1. Cell Culture and Osteogenic Induction

The mouse embryo fibroblasts cell line C3H10 T1/2 clone 8 (obtained from the Cell Resource Center, Peking Union Medical College) was cultured at 37 °C, 5% CO_2_ in high glucose DMEM (Hyclone, Logan, UT, USA) supplemented with 10% FBS (Gibco, Grand Island, NY, USA) and 1% antibiotic, and the culture medium was replaced every 2 days. Osteogenic differentiation was induced in complete medium with 10^−8^ M dexamethasone (Sigma-Aldrich, St. Louis, MO, USA), 10^−2^ M β-glycerophosphate disodium salt hydrate (Sigma-Aldrich), and 5 × 10^−5^ M ascorbic acid (Sigma-Aldrich).

### 2.2. Cell Proliferation Assay

Cell proliferation was analyzed using the CCK-8 cell proliferation and cytotoxicity assay kit according to the manufacturer’s instructions (Solarbio Biotechnology Co., Ltd., Beijing, China). Briefly, cells were seeded in a 96-well plate at a density of 5 × 10^3^ cells/well and cultured with complete DMEM. After 24 h, they were incubated with 0–10^−4^ M of VK_2_ (Sigma-Aldrich) for 6, 12, 24, and 48 h. CCK-8 solution was added to the 96-well plate and incubated for 1 h. Then, the 96-well plate was read with a multimode reader (BioTek, Winooski, VT, USA) to obtain the absorbance at 450 nm, and to calculate the cell viability.

### 2.3. Sample Preparation and RNA Sequencing (RNA-Seq)

Cells were treated with 0 or 10^−5^ M concentrations of VK_2_ in osteogenic medium for 7 days. Total RNA of cells was extracted using Trizol (Invitrogen, Carlsbad, CA, USA) and used for library construction. RNA quality was determined by Agilent 2100 Bioanalyzer (Agilent Technologies, Palo Alto, CA, USA) and NanoDrop 2000 (Thermo Fisher Scientific, Waltham, MA, USA). Then, Oligo (dT) beads were used to enrich eukaryotic mRNA, and the mRNA was broken into short fragments by adding fragmentation buffer. The target mRNA fragments were reversely transcribed into cDNA using random primers. The second cDNA strand was synthesized by adding buffer solution, dNTPs, RNase H and DNA polymerase I, and the double-stranded cDNA was purified. The cDNA fragments were end-repaired, poly (A) added, and PCR amplification. DNA library quality was determined by Agilent 2100 Bioanalyzer (Agilent Technologies, Palo Alto, CA, USA) and ABI StepOnePlus RealTime PCR System (Applied Biosystems, Waltham, MA, USA). After library construction, the DNA library was sequenced using MGISEQ T7 sequenator (Huada Smart Manufacturing Technology Co., Ltd., Shenzhen, China). High-quality clean reads were obtained by removing reads with low quality, linker contamination, and unknown base N content greater than 5%. Clean reads were mapped to the reference genome using HISAT2 (version 2.2.1) and mapped to the reference sequence using Bowtie2 (version 2.4.5) [17]. Then, RSEM was used to calculate the expression levels of genes and transcripts, and cor function, hclust function, and princomp function of R language was used to perform correlation analysis, hierarchical clustering analysis, and principal component analysis (PCA). DEseq2 software was used to perform RNAs differential expression analysis and the parameter of fold change ≥ 2.00 and adjusted *p*-value ≤ 0.05 were considered differentially expressed transcripts.

### 2.4. Bioinformatics Analysis

Gene ontology (GO) functional analyses, Kyoto Encyclopedia of Genes and Genomes (KEGG) pathway enrichment analyses was performed using R package clusterProfiler. STRING (version 11.5) was used to construct the network of protein–protein interaction (PPI) [18]. Then, degree algorithm of Cytoscape software was used to identify hub genes [19].

### 2.5. Small Interfering RNA (siRNA) Assay

The double stranded small interfering RNA (siRNA) was synthesized by Shanghai GenePharma Co., Ltd. (Shanghai, China). The primer sequences of IL-6 were as follows: 5′-CCUCUGGUCUUCUGGAGUATT-3′ and 5′-UACUCCAGAAGACCAGAGGTT-3′, and the primer sequences of Bcl-6 were as follows: 5′-GAUGAGAUUGCCCUGCAUUTT-3′ and 5′-AAUGCAGGGCAAUCUCAUCTT-3′. Cells were incubated in 6-well plates. After reaching 80% confluence, cells were transfected with 50 nM siRNA or negative control (NC) siRNA using LipoRNAiMAX Transfection Reagent (Mei5 Biotechnology Co., Ltd., Beijing, China) in Opti-MEM (Gibco, Grand Island, NY, USA) following the product manuals. After transfection for 6 h, the culture medium was replaced with osteogenic differentiation medium and the culture was continued for 3 days.

### 2.6. Construction of Recombinant Plasmids and Transfection Assay

The genomic DNA of IL-6 and Bcl-6 were synthesized by Beijing New Times Zhonghe Technology Co., Ltd. (Beijing, China). The IL-6 or Bcl-6 DNA was inserted into the mammalian expression vector, pcDNA3.1-EGFP-C (You Bio Co., Ltd., Changsha, China) and the sequences were verified. The IL-6 or Bcl-6 expression vector was transfected into C3H10 T1/2 clone 8 cells using the Lipo3000 Transfection Reagent (Mei5 Biotechnology Co., Ltd., Beijing, China) according to the product manuals. Transfected C3H10 T1/2 clone 8 cells were cultured in osteogenic differentiation medium for 3 days.

### 2.7. Alkaline Phosphatase (ALP) Staining and Activity

Cells were seeded in 6-well plates in complete medium. After the cells reached 80~90% confluence, the medium was replaced by the osteogenic medium with different concentrations of VK_2_ for 7 days. Then, the cells were fixed for 20 min with 4% paraformaldehyde, and ALP staining was performed using the BCIP/NBT alkaline phosphatase chromogenic kit following the manufacturer’s instructions (Beyotime Biotechnology, Beijing, China). Cells were observed under a light microscope (Nikon, Tokyo, Japan) after staining by neutral red staining solution (Beyotime Biotechnology) for 5 min.

After treatment with VK_2_ for 7 days, the cells were washed with PBS and lysed with RIPA lysis buffer (Beyotime Biotechnology). Cell lysates were analyzed for protein concentration using the BCA protein concentration assay kit (Beyotime Biotechnology). Then, ALP activity was measured using an ALP assay kit according to the manufacturer’s instructions (Nanjing Jiancheng Bioengineering Institute, Nanjing, China).

### 2.8. Real-Time PCR Analysis

For analysis of gene expression, total RNA was extracted from cultured cells using the EASY spin Plus Bone Tissue RNA Kit (Aidlab Biotechnologies Co., Ltd., Beijing, China). The cDNA was generated using a reverse transcription kit (Mei5 Biotechnology Co., Ltd., Beijing, China). An SYBR Premix EsTaq reagent (Mei5 Biotechnology Co., Ltd., Beijing, China) was used for real-time PCR by the StepOnePlus real-time PCR system (Applied Biosystems, Waltham, MA, USA). The relative expression was normalized to GAPDH. The primers are shown in Supplementary Table S1.

### 2.9. Western Blot Analysis

Proteins were extracted from the cells using RIPA lysis buffer and separated by sodium dodecyl sulfate-polyacrylamide gel electrophoresis, and electro-transferred on polyvinylidene fluoride (PVDF) membranes. Then, the membranes were blocked using 5% skimmed milk for 1 h at room temperature, and were incubated overnight at 4 °C with RUNX2 (CST, No. 8486), STAT1 (CST, No. 14994), and GAPDH (CST, No. 5174). The membranes were washed with TBST three times for 10 min each time, and were incubated 1 h at room temperature with second antibody, washed three times with TBST. Finally, the membranes were exposed to Odyssey Clx (LI-COR, Lincoln, NE, USA) to obtain the quantities of the proteins.

### 2.10. Statistical Analysis

All of the results are presented as the mean ± SD. One-way ANOVA followed by Tukey–Kramer test was performed for multiple comparisons. SAS 9.4 (SAS Institute, Cary, NC, USA) was used for statistical analysis.

## 3. Results

### 3.1. Effects of VK_2_ on the Proliferation of C3H10 T1/2 Clone 8 Cells

**Figure 1:**
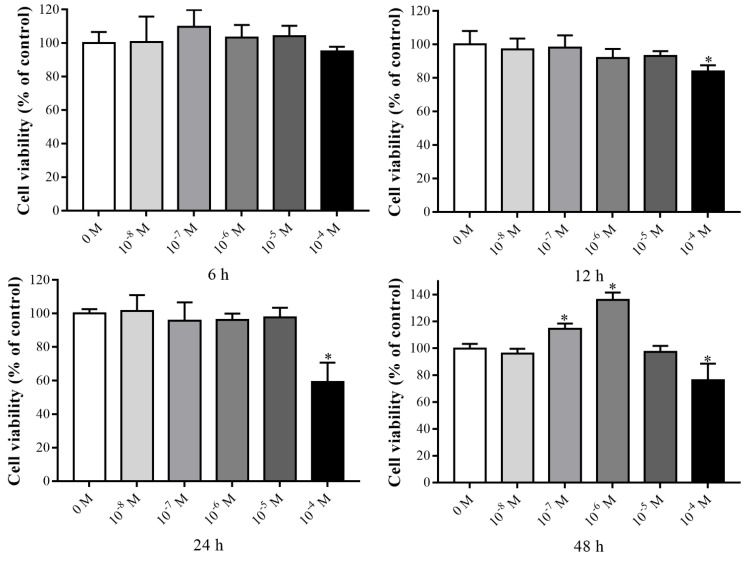
Cell viability effect of VK_2_ on C3H10 T1/2 clone 8 cells. The data are presented as the mean ± SEM; * denotes a significant difference with respect to the control group (*p* < 0.05).

We performed a cell viability test to identify the ability of VK_2_ to affect the proliferation of C3H10 T1/2 clone 8 cells, the cells were treated by different doses and different incubation times. Cells were cultured for 12, 24, and 48 h, 10^−4^ M VK_2_ significantly inhibited cell proliferation (Figure 1). However, at the incubation time of 48 h, cell viability was increased following the treatment of cells with 10^−7^ M or 10^−6^ M VK_2_.

### 3.2. VK_2_ Promoted Osteogenic Differentiation in C3H10 T1/2 Clone 8 Cells

**Figure 2:**
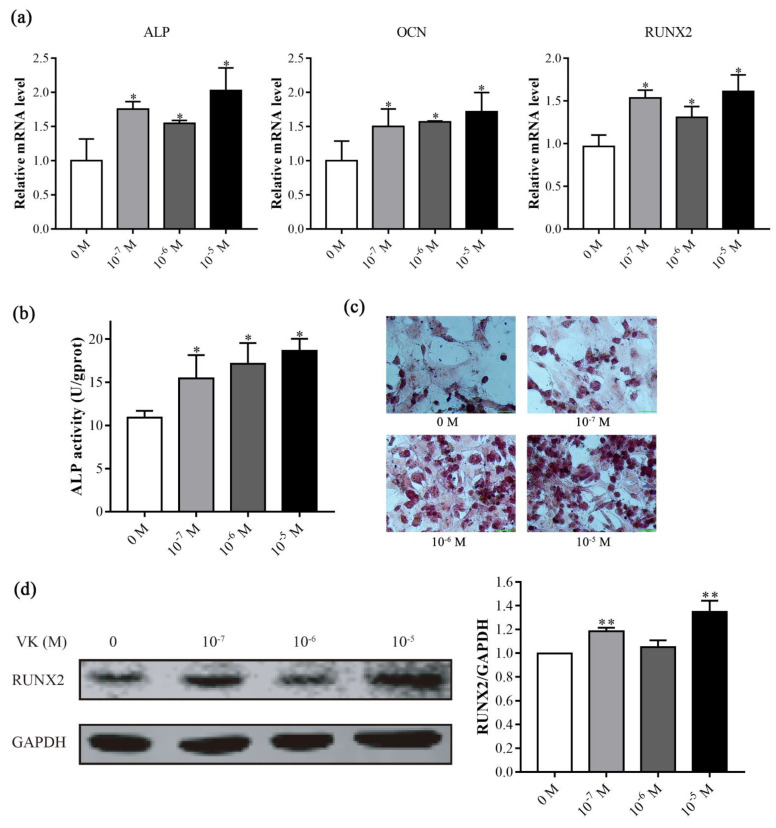
VK_2_ promoted osteogenesis in C3H10 T1/2 clone 8 cells in vitro. (**a**) The mRNA levels of ALP, OCN, and RUNX2 were detected after VK_2_ treatment for 7 days. (**b**,**c**) The effect of VK_2_ on ALP during osteoblastogenesis, as determined by ALP activity (**b**) and ALP staining (**c**). (**d**) The protein level of RUNX2 after VK_2_ treatment for 7 days. The data are presented as the mean ± SEM; *, ** denotes a significant difference with respect to the control group (*p* < 0.05).

We evaluated the effects of VK_2_ on osteoblast differentiation in C3H10 T1/2 clone 8 cells. VK_2_ at 10^−7^–10^−5^ M significantly increased the expression of osteoblast differentiation genes, ALP, OCN, and RUNX2 (Figure 2a). Moreover, ALP activity was strongly induced by different concentrations of VK_2_ (Figure 2b), which was further confirmed by ALP staining (Figure 2c). We further detected the protein level of the key transcription factor RUNX2, and found that VK_2_ at 10^−7^ and 10^−5^ M significantly increased the level of RUNX2 protein (Figure 2d). We used 10^−5^ M of VK_2_ for the following experiments. These results indicated that VK_2_ could improve osteoblastic osteogenic differentiation in vitro.

### 3.3. RNA-Seq Analysis of C3H10 T1/2 Clone 8 Cells upon VK_2_ Treatment

**Figure 3:**
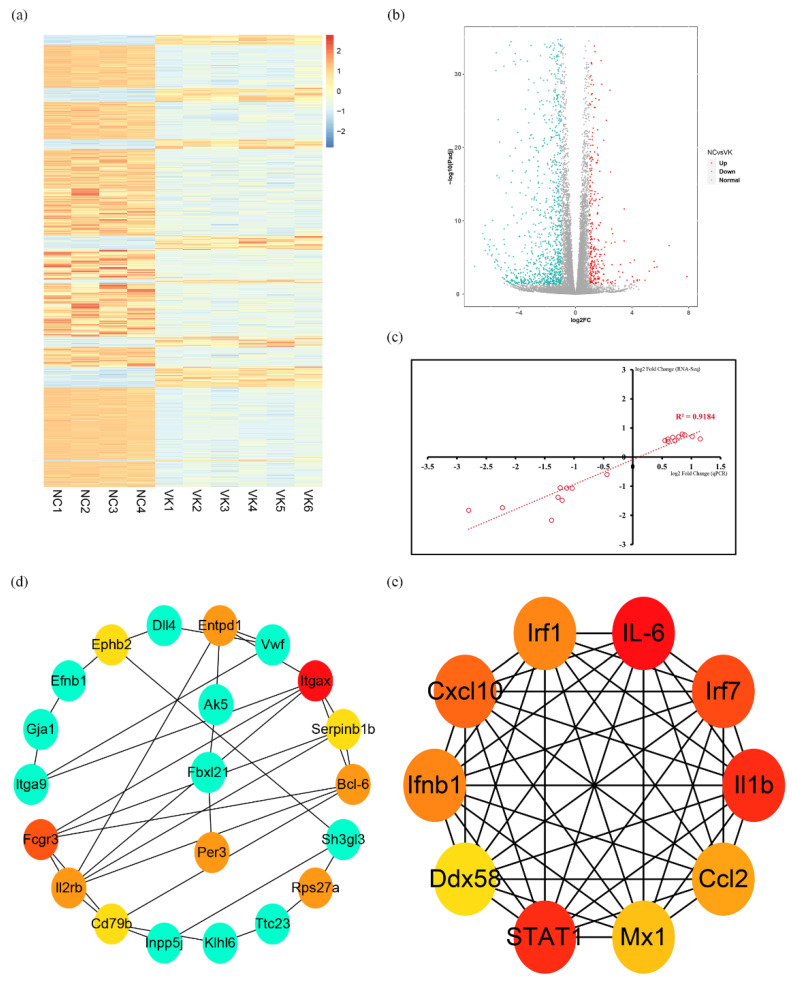
DEGs profiling by RNA-seq. (**a**) Heatmap of RNA-seq. (**b**) Volcano plots of RNA-seq. (**c**) Reliability verification of RNA-seq data. (**d**) Up-top 10 in network string–string interactions ranked by Degree method. (**e**) Down-top 10 in network string–string interactions ranked by Degree method.

**Figure 4:**
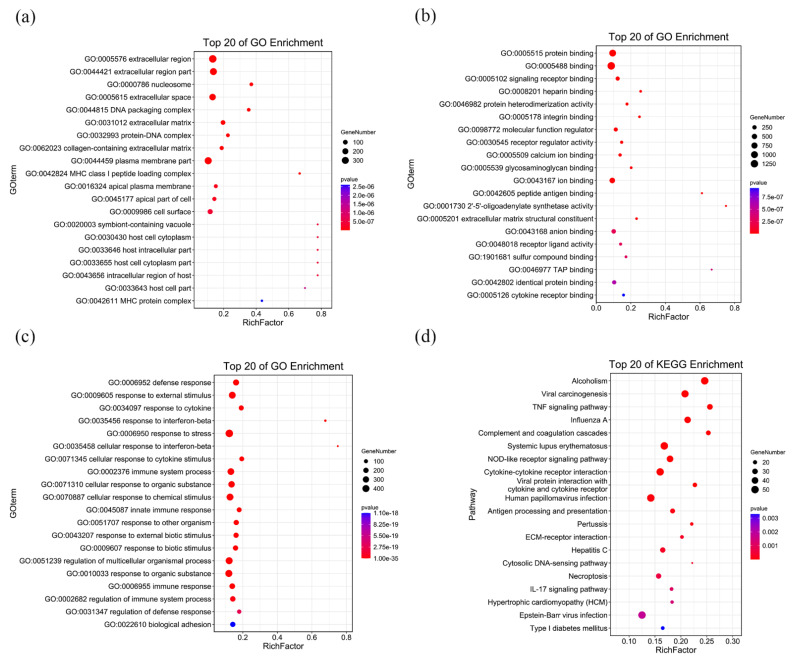
GO analysis and KEGG function enrichment analysis. (**a**) GO function enrichment-cell component. (**b**) GO function enrichment-molecular function. (**c**) GO function enrichment-biology process. (**d**) KEGG function enrichment analysis.

We investigated the mechanism of VK_2_ promoting osteogenic differentiation using an RNA-seq analysis. The sample parameters are shown in Supplementary Table S2. The cluster heat map analysis showed good intra-group repeatability (Figure 3a). Analysis revealed that 1662 annotated or potential differentially expressed genes (DEGs) were upregulated or downregulated. Among them, 1348 DEGs showed a decrease in expression, while the remaining DEGs (314) were highly upregulated in the VK_2_ group compared with the NC group (Figure 3b). The validation of RNA-seq demonstrated the reliability of the results (Figure 3c). GO analysis showed that total 1212 GO terms were significantly enriched, including 53 terms of cellular component (CC), 95 terms of molecular function (MF), and 1064 terms of biological process (BP). The top 20 enriched GO terms of CC, MF, BP are shown in Figure 4a–c. KEGG pathway showed that 46 pathways were significantly enriched, and the top 20 enriched pathways were shown in Figure 3d. Next, we analyzed the DEGs using STRING 11.5 and Cytoscape software to identify the interaction among DEGs and search for downstream target molecules. The top 10 hub genes within the 314 up-DEGs are Itgax, Fcgr3, Entpd1, Rps27a, Per3, Bcl-6, Il2rb, Ephb2, Serpinb1b, and Serpinb6c (Figure 3d). The top 10 hub genes within the 1348 down-DEGs are IL-6, IL1b, STAT1, IRF7, Cxcl10, IFNB1, IRF1, Ccl2, Mx1, and Ddx58 (Figure 3e).

### 3.4. VK_2_ Promotes Osteogenic Differentiation by Upregulating Bcl-6

**Figure 5:**
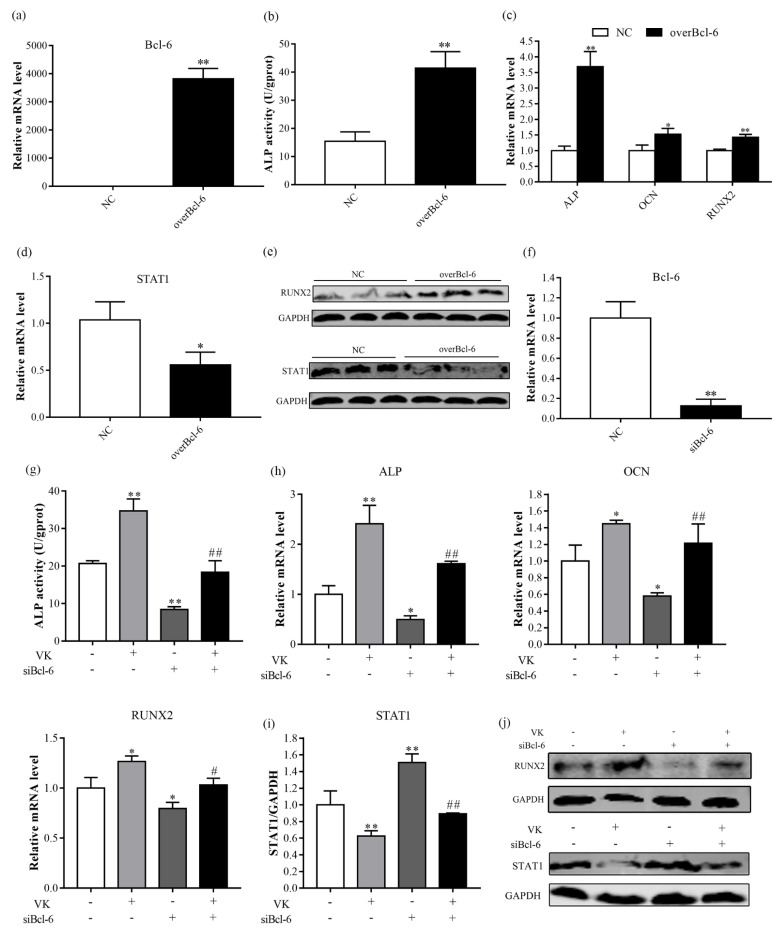
VK_2_ promoted osteogenesis by inhibiting STAT1 via Bcl-6 in C3H10 T1/2 clone 8 cells. (**a**) Bcl-6 overexpression efficiency. (**b**) ALP activity after Bcl-6 overexpression. (**c**,**d**) The mRNA levels of ALP, OCN, RUNX2 (**c**), and STAT1 (**d**) after Bcl-6 overexpression. (**e**) The protein levels of RUNX2 and STAT1 after Bcl-6 overexpression. (**f**) Knockdown efficiency of Bcl-6. (**g**) ALP activity after Bcl-6 knockdown during VK_2_ induced osteoblastic differentiation. (**h**,**i**) The mRNA levels of ALP, OCN, RUNX2 (**h**), and STAT1 (**i**) after Bcl-6 knockdown during VK_2_ induced osteoblastic differentiation. (**j**) The protein levels of RUNX2 and STAT1 after Bcl-6 knockdown during VK_2_ induced osteoblastic differentiation. The data are presented as the mean ± SEM; *, ** denotes a significant difference with respect to the control group (*p* < 0.05); #, ## denotes a significant difference with respect to the siBcl-6 group (*p* < 0.05).

A previous study reported that Bcl-6 plays a crucial role in osteoblast activation through STAT1 inhibition [20]. Given that VK_2_ could increase mRNA level of Bcl-6 and decrease mRNA level of STAT1, this raised the possibility that VK_2_ improves osteogenic activity by inhibiting STAT1 through Bcl-6 regulation. To verify this hypothesis, we utilized overexpression of pcDNA3.1-EGFP-C-Bcl-6, and the transfection efficiency was measured at mRNA level (Figure 5a). Overexpression of Bcl-6 increased ALP activity, mRNA levels of ALP, OCN, and RUNX2, and protein level of RUNX2 (Figure 5b–e). Moreover, Bcl-6 overexpression reduced expression of STAT1 (Figure 5d,e). In contrast to Bcl-6 overexpression, knockdown of Bcl-6 suppressed osteogenic differentiation and increased expression of STAT1 (Figure 5g–j). However, VK_2_ reversed the inhibition of osteogenesis caused by knockdown of Bcl-6 (Figure 5g,h,j) and the expression of STAT1 (Figure 5i,j).

### 3.5. VK_2_ Promotes Osteogenic Differentiation by Downregulating IL-6

**Figure 6:**
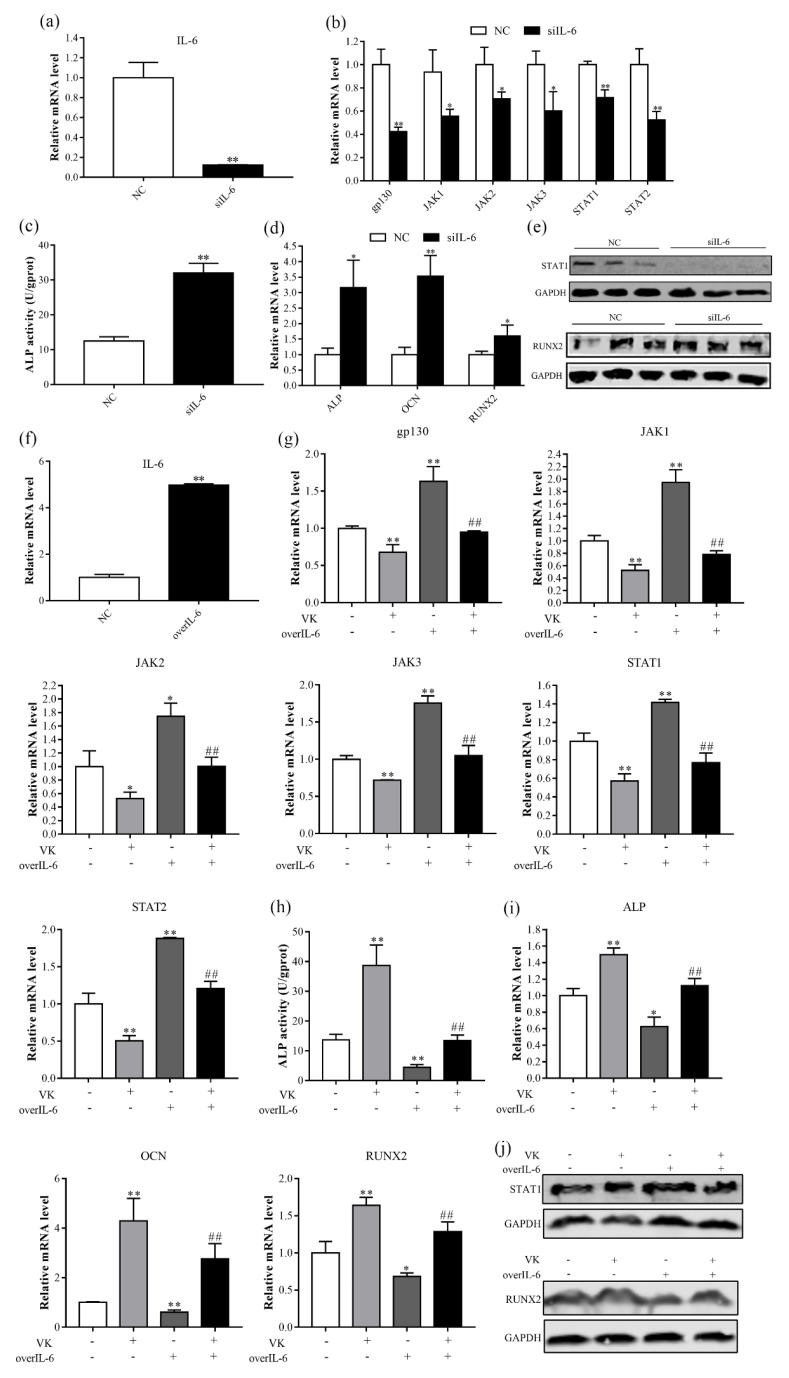
VK_2_ promoted osteogenesis by inhibiting STAT1 via IL-6 in C3H10 T1/2 clone 8 cells. (**a**) Knockdown efficiency of IL-6. (**b**,**d**) The mRNA levels of gp130, JAK1, JAK2, JAK3, STAT1, STAT2 (**b**), and ALP, OCN, and RUNX2 (**d**) after IL-6 knockdown. (**c**) ALP activity after IL-6 knockdown. (**e**) The protein levels of RUNX2 and STAT1 after IL-6 knockdown. (**f**) IL-6 overexpression efficiency. (**g**,**i**) The mRNA levels of gp130, JAK1, JAK2, JAK3, STAT1, STAT2 (**g**), and ALP, OCN, and RUNX2 (**i**) after IL-6 overexpression during VK_2_ induced osteoblastic differentiation. (**h**) ALP activity after IL-6 overexpression during VK_2_ induced osteoblastic differentiation. (**j**) The protein levels of RUNX2 and STAT1 after IL-6 overexpression during VK_2_ induced osteoblastic differentiation. The data are presented as the mean ± SEM; *, ** denotes a significant difference with respect to the control group (*p* < 0.05); ## denotes a significant difference with respect to the IL-6 group (*p* < 0.05).

The binding of IL-6 to its receptors subsequently activates the JAK/STAT signaling pathway [21]. VK_2_ significantly decreased mRNA level of IL-6, we speculate that VK_2_ improved osteoblast differentiation through the IL-6-mediated JAK/STAT signaling pathway. Our results showed that knockdown of IL-6 decreased mRNA levels of gp130, JAK1, JAK2, JAK3, STAT1, and STAT2, and protein level of STAT1 (Figure 6b,e). In addition, knockdown of IL-6 improved ALP activity, mRNA levels of ALP, OCN, and RUNX2, and protein level of RUNX2 (Figure 6c–e). In contrast, overexpression of IL-6 enhanced JAK/STAT signaling pathway activity and decreased osteogenic differentiation (Figure 6g–j), which were reversed in the presence of VK_2_.

## 4. Discussion

Decreased bone formation disrupts the homeostasis of bone turnover, leading to increased risk of fractures and is the root cause of osteoporosis [22]. We have previously reported that VK_2_ improved the serum bone transformation status, bone microarchitecture, and gene abundance associated with bone formation in ovariectomized mice in the previous study [13]. Previous studies reported that VK_2_ induced osteogenic differentiation via activating Wnt pathways, promoting autophagy, and inhibiting miR-133a expression [8,23,24]. It is possible that VK_2_ regulates bone metabolism through multiple mechanisms. In this study, we found that VK_2_ function also involves the regulation of Bcl-6 and IL-6 expression.

ALP is a key enzyme in the formation of extracellular matrix of osteoblasts, which has phosphatase activity and can hydrolyze naturally occurring phosphorous substrates, release free phosphate ions, and participate in the mineralization deposition of extracellular matrix [25]. ALP expression begins to increase during the late proliferation stage through differentiation followed by a decline in the late differentiation stage. As an important signal of osteoblast differentiation, ALP is widely used as an early marker of the differentiation process [26,27]. OCN is specifically produced by osteoblasts and is the most abundant non-collagenous protein in bone, and can combine calcium and phosphorus ions to form mature mineralized matrix, and has been a specific indicator for evaluating osteoblast activity [26,28,29]. Our results showed that VK_2_ increased the ALP activity and mRNA levels of ALP and OCN in C3H10 T1/2 clone 8 cells. Consistent with our finding, Cui et al. [23] found that VK_2_ increased ALP activity of periodontal ligament stem cells. Moreover, Li et al. [8] found that VK_2_ increased ALP activity of MC3T3-E1 osteoblasts. As an osteoblast specific transcription factor, RUNX2 controls skeletal development by regulating the differentiation of chondrocytes and osteoblasts and the expression of many extracellular matrix protein genes during chondrocyte and osteoblast differentiation [28,30]. VK_2_ increased the RUNX2 expression of mRNA and protein in the present study, which is in agreement with the results of Zhang et al. [24], that reported similar results in human bone marrow stromal cells. Our results indicate that VK_2_ promotes osteogenic differentiation in C3H10 T1/2 clone 8 cells.

Osteoblast differentiation involves complex spatiotemporally regulated molecular interaction among multiple transcription factors [31,32]. The goal of a good RNA-seq experiment is not to reach definitive conclusions, but to generate new, testable hypotheses [33]. As a research method in molecular biology, RNA-seq has been widely applied to a very diverse set of problems in skeletal biology. In the present study, we performed the RNA-seq analysis to explore the mechanism that VK_2_ improves osteoblast differentiation. The qRT-PCR analysis showed that the trends for 19 genes were consistent with the RNA-seq results (Figure 2c). These results suggest that the RNA-seq results were reliable. By analysis of differential genes using STRING database and Cytoscape software, the top 10 hub genes that were upregulated or downregulated were identified.

Bcl-6 is a transcriptional repressor that is mainly involved in cell activation, differentiation, and proliferation [34]. Miyauchi et al. [35] showed that Bcl-6-overexpression inhibited osteoclastogenesis in vitro, whereas Bcl-6-deficient mice showed accelerated osteoclast differentiation, which were mediated by Bcl-6 directly targeting osteoclast differentiation related molecules, such as NFATc1, cathepsin K, and dendritic cell-specific transmembrane protein (DC-STAMP). Fujie et al. [20] indicated that STAT1 is a direct target of Bcl-6, and STAT1 deletion can restore some bone mass and osteoblastic parameters observed in Bcl-6-deficient mice. We noticed in the present study that overexpression of Bcl-6 resulted in significant elevation of osteogenic differentiation and decrease of STAT1 mRNA and protein expression in C3H10 T1/2 clone 8 cells, which was similar to the previous study [20]. In contrast, siBcl-6 decreased osteogenic differentiation and increased the STAT1 mRNA and protein expression. In addition, VK_2_ significantly increased the mRNA level of Bcl-6, and decreased the mRNA level of STAT1, and alleviated the inhibition of siBcl-6 on osteogenic differentiation. Therefore, the regulation of osteogenic metabolism by VK_2_ may be through the Bcl-6/STAT1 axis.

The JAK/STAT signaling pathway is an important downstream mediator for a variety of cytokines, hormones, and growth factors [36]. IL-6 is an inflammatory cytokine of IL-6 family and can regulate pleiotropic, haematopoiesis, acute phase response, and lymphoid differentiation by JAK/STAT pathway [37]. Previous study found that IL-6 enhances oteoclastogenesis and JAK2 activation in osteocyte-like MLO-Y4 cells [38]. In the present study, we found that knockdown of IL-6 inhibits mRNA levels of genes related to the JAK/STAT pathway, which was consistent with the previous study [37]. In addition, we indicated that knockdown of IL-6 decreased STAT1 protein expression and increased osteogenic differentiation, which is mostly due to STAT1 inhibition of the nuclear localization of RUNX2 by interacting with RUNX2 in the cytoplasm, thereby inhibiting osteoblast differentiation [39]. These findings suggested that osteoblasts with impaired IL-6 promote osteogenic differentiation in vitro. In contrast to IL-6 knockdown, overexpression of IL-6 increased mRNA levels of genes related to the JAK/STAT pathway and STAT1 protein expression and decreased osteoblast differentiation. Furthermore, VK_2_ reversed the indicators induced by overexpression of IL-6. The results suggest that VK_2_ improves osteogenic differentiation in C3H10 T1/2 clone 8 cells possibly through a decrease in expression of IL6/JAK/STAT pathway, which provides novel insights into a fundamental regulatory role of VK_2_ in regulating bone metabolism.

## 5. Conclusions

In summary, the present study indicated that VK_2_ could promote osteogenic differentiation in C3H10 T1/2 clone 8 cells via Bcl-6/STAT axis and IL-6/JAK/STAT signaling pathway.

## Data Availability

The data that support the findings of this study are available from the corresponding author upon reasonable request.

## Supplementary Materials

The following supporting information can be downloaded at: https://www.mdpi.com/article/10.3390/nu14142934/s1. Table S1: Primers used for real-time PCR experiments; Table S2: The sample parameters.
