# Supplementary material for: Vitamin K2 Improves Osteogenic Differentiation by Inhibiting STAT1 via the Bcl-6 and IL-6/JAK in C3H10 T1/2 Clone 8 Cells
Source: Nutrients. 2022 Jul 18;14(14):2934. doi: 10.3390/nu14142934 (PMC9316273; doi:10.3390/nu14142934)
Supplement: Supplementary file 1 [file nutrients-14-02934-s001.zip › nutrients-1796980-supplementary.pdf]

Supplementary Table S1. Primers used for real-time PCR experiments.

Gene		Primer sequence
GAPDH	Forward	5’- CCCACTCTTCCACCTTCGAT-3’
	Reverse	5’- CTTGCTCAGTGTCCTTGCTG-3’
ALP	Forward	5’- GAAGGCTCTCTTCACTCCAAGA-3’
	Reverse	5’- GGCGACAGGTGAAGAAACAG-3’
OCN	Forward	5’-GAACAGACAAGTCCCACACAGC-3’
	Reverse	5’-TCAGCAGAGTGAGCAGAAAGAT-3’
RUNX2	Forward	5’-GCACCGACAGCCCCAACTT-3’
	Reverse	5’-CCACGGGCAGGGTCTTGTT-3’
Herc6	Forward	5’-GTATTCCTGCGGGGACAACA-3’
	Reverse	5’-TCCCCAAGCGAAGACCTTTC-3’
IL6	Forward	5’-CCCCAATTTCCAATGCTCTCC-3’
	Reverse	5’-GGATGGTCTTGGTCCTTAGCC-3’
Stat1	Forward	5’-CTTTGTTCCCTTTCAGACCACC-3’
	Reverse	5’-GTACCACAGGATAGACGCCC-3’
Socs2	Forward	5’-CAGCTGGACCGACTAACCTG-3’
	Reverse	5’-TGAACAGTCCCATTCCGTGG-3’
Irf7	Forward	5’-GGCAAGAGAAAATGCTGGGC-3’
	Reverse	5’-CGGCCCTTGTACATGATGGT-3’
Gbp3	Forward	5’-GAATGAGGCTCCCCACAGAC-3’
	Reverse	5’-CAGCCTGGCAGTGACGAATA-3’
Stat2	Forward	5’-AGCAGCCATCAGAGTCAGATTT-3’
	Reverse	5’-CCCAAGAGTCCATCCGCATC-3’
Gdpd2	Forward	5’-CTGGGATGGCTGTACATCGG-3’
	Reverse	5’-GCCCAGGAGCAATAGCAAGG-3’
Ddx58	Forward	5’-CACTGTCCTTGGAGACGCTT-3’
	Reverse	5’-GGTTCTGAACTCCGCTCACA-3’

Morc4	Forward	5’-AGCACATGGGTTATCGCCAA-3’
	Reverse	5’-TGGCCAAAGCCCTTTCTAGG-3’
Fcgr3	Forward	5’-TCCCTAGTGATGTGCCTCCT-3’
	Reverse	5’-AGTAATCCCTCGGGGTTTGAA-3’
Bcl6	Forward	5’-GAGTCGGGACATCTTGACGG-3’
	Reverse	5’-TCCAGGAGGATGCAAAACCC-3’
Hyal1	Forward	5’-TGCCTGACGGAGTATGGAGT-3’
	Reverse	5’-GCAGACCACCAAATACGGGT-3’
Hoxc13	Forward	5’-GACAAGTACCCAGAGCCGTC-3’
	Reverse	5’-AGCCTTCAACAGGGATGAGC-3’
Pfn2	Forward	5’-CCAGAGCATCACGCCAGTAG-3’
	Reverse	5’-ACGTTGTATGTTGGCTCCCC-3’
Ttc23	Forward	5’-CAAGCCCTAGGACTCTACGC-3’
	Reverse	5’-GGTACGTTTCTGCCACCTCA-3’
Peli3	Forward	5’-TGGGCTACAATGGGTGTCTG-3’
	Reverse	5’-GGCCAATCTTGGAGACGAGT-3’
Mst1	Forward	5’-CTGATTTGCCTGCCTCCTGA-3’
	Reverse	5’-CAGTCCCTGGGTGCATAT-3’
Lpin1	Forward	5’-CCAGCCCCAGTCCTTCAG-3’
	Reverse	5’-TTTGCAGCCTGTGGCAATTC-3’

Supplementary Table S2. The sample parameters.

Sample Name	Raw Reads	Raw Base	Clean Reads	Clean Base	Q20	Q30	GC content, %
NC1	96.46M	14.47G	93.49M	14.02G	98.60%	95.43%	51.01%
NC2	98.07M	14.71G	94.72M	14.21G	98.62%	95.50%	51.06%
NC3	84.59M	12.69G	81.77M	12.27G	98.48%	95.06%	51.27%
NC4	111.40M	16.71G	106.90M	16.03G	98.69%	95.72%	50.96%
VK1	98.06M	14.71G	93.83M	14.08G	98.63%	95.55%	51.47%

VK2	83.16M	12.47G	79.33M	11.90G	98.05%	93.90%	51.46%
VK3	99.03M	14.85G	94.38M	14.16G	98.64%	95.60%	51.14%
VK4	64.61M	9.69G	64.01M	9.60G	97.33%	92.17%	51.36%
VK5	97.82M	14.67G	93.07M	13.96G	98.74%	95.84%	51.41%
VK6	100.55M	15.08G	95.41M	14.31G	98.72%	95.83%	51.48%
